# The Alexafication of Adult Social Care: Virtual Assistants and the Changing Role of Local Government in England

**DOI:** 10.3390/ijerph18020812

**Published:** 2021-01-19

**Authors:** James Wright

**Affiliations:** The Alan Turing Institute, London NW1 2DB, UK; j.wright@turing.ac.uk

**Keywords:** care, Alexa, technology, smart speaker, telecare, Amazon, England, virtual assistant

## Abstract

Voice controlled virtual assistants, delivered via consumer devices such as smart speakers and tablets, are being trialled by local authorities across England as a convenient and low-cost supplement or potential alternative to “traditional” telecare. Few papers have explored this increasingly widespread phenomenon, despite its growing importance. This article looks at choices by some local authorities to trial Alexa, within the context of the ongoing care crisis in England, with councils facing depleted funds, a lack of expert guidance on care technologies, and an increasingly complex and fragmented care technology marketplace. It draws on interviews with managers from eight English local authorities involved in the commissioning and trialling of technologies for adult social care to examine how and why virtual assistants are being implemented, and what implications their use might hold for care. Scaling up the application of such technologies could shift the role of local authorities towards one of an app developer and data broker, while generating considerable risks of reliance on the precarious technological infrastructure of global corporations that may have little interest in or sensitivity towards local care concerns. The findings suggest an urgent need for a national social care technology strategy and increased support for local authorities.

## 1. Introduction

At the 2018 International Technology Enabled Care (ITEC) Conference, perhaps the most important venue for the exhibition of new adult care technologies in the U.K., all of the usual companies were present: long-established telecare giants Tunstall and Legrand, as well as various smaller care device companies and new start-ups offering a range of specialist care equipment and services. But one company was not showing off any product in particular despite renting a booth in the exhibition hall. Amazon Web Services’ stall seemed more a symbolic staking of a claim in the U.K. care technology market than an attempt to sell a specific product.

Voice controlled virtual assistants, such as Amazon’s Alexa, Apple’s Siri, and Google’s Assistant, delivered through smart speakers such as Amazon’s Echo and Dot, and Google’s Home, and through smartphones and tablets, have become increasingly attractive among English local authorities as a possible near-future alternative to or replacement for “traditional” telecare devices and services. While the latter have typically consisted of simple pendant alarms connected via fixed analogue phone lines with 24/7 call centres, voice controlled virtual assistants can be used not only to initiate phone calls, but also to connect users to a wide range of other online services, as well as offering control over linked digital networked devices, such as “smart” lights or thermostats. In November 2020, Amazon released the Alexa Care Hub (www.amazon.com/Alexa-Care-Hub/), a service that enables a user to remotely “check in on” people they care for. Alexa is already used in British healthcare: in 2019, the National Health Service (NHS) contracted with Amazon to enable Alexa to answer health-related questions, raising questions of privacy and how healthcare data would be used [1,2,3].

However, despite the rapidly growing use of virtual assistants in the delivery of health and social care, much social scientific work on the growing application of artificial intelligence (AI) to public services has focused on algorithmic decision-making systems [4], datafication [5], a “new public analytics” paradigm [6], and an emerging “digital welfare state” in which governments are increasingly privatising and automating welfare management [7]. Relatively few studies have examined the use of these technologies in the delivery of adult social care. One such study identified some benefits of using Amazon’s Echo with Alexa for relational autonomy among care recipients with cognitive impairments and their caregivers [8], while another has found that the majority of carers studied had positive experiences of using various assistive technologies in dementia care at home, including voice controlled virtual assistants, although it also found that in some cases people with dementia forgot how to operate Alexa [9]. Hamblin has examined some of the challenges involved in upgrading and potentially replacing elements of telecare with new technology-enabled care services as a result of the planned digital switchover in 2025 [10].

The present study aims to build on this emerging literature by taking a broader view of the implications of the use of such devices for the adult social care system at large, in order to understand how virtual assistant technologies may transform not only the delivery of care, but also the role of the local authorities responsible for it. In so doing, I hope to contribute to a currently neglected aspect of the larger ongoing debate about how global corporations are extending their influence over public health and social care systems—and, in turn, over aspects of local government—through the introduction of new digital technologies, within the context of the ongoing commodification and fragmentation of care in England and beyond. To address these issues, the paper focuses on a group that is frequently overlooked in studies of care technologies: the commissioners and decision-makers working in the adult social care departments of local government who often act as gatekeepers for public spending on their adoption—in England as in many other welfare states.

This article draws on semi-structured interviews conducted at the start of 2020 with employees from eight English local authorities (LAs) who were involved in commissioning, trialling, and working with digital technologies used in the provision of adult social care (ASC), including voice controlled virtual assistants. It considers why many LAs in England and beyond are turning to the use of smart speakers and other consumer electronic devices, and what implications such a shift may hold for the future role of LAs in relation to their statutory responsibility to provide ASC, as well as what risks greater adoption of such technologies may create for England’s ASC system.

### The Care Technology Landscape in England

The delivery of ASC in England is the statutory responsibility of the country’s 152 LAs. The cost of care provision is funded partly by an annual service budget from central government, and partly by other sources of funding including each LA’s central budget, and the NHS budget. LA commissioners are responsible for contracting with care providers for goods and services, such as residential care, telecare equipment and services, and other care technologies. The provision of many of these services is outsourced to private companies, although some are delivered in-house, for example through council-run telecare call centres or residential care facilities. ASC is generally not provided free of charge in England until the citizen paying for the ASC services they require has run out of their own money, at which point the LA will step in. However, some LAs partially or fully fund services such as telecare for all residents who require them.

In the early 2000s, the U.K. was a European leader in care technologies. Fuelled by a combination of fears about the predicted future cost of elder care, and a technocratic New Labour administration willing to invest strategically in public services and emerging digital technologies, the U.K. government provided GBP 132 million in funding for the widespread implementation of telecare between 2006–2008, mainly through a Preventative Technology Grant to LAs in England [11]. A European Commission report in 2010 described the promotion of telecare by the U.K. government as “probably the most comprehensive example internationally to date” [12]. Telecare remains the most widely used care technology deployed by LAs in England.

However, despite significant ongoing investments since then at the national and EU level in the development of innovative new technologies of elder care, central U.K. government investment in the implementation of these technologies waned in the 2010s, and LAs were largely left to navigate the increasingly complex and fragmented care tech marketplace without recourse to central state financial support or advice [11]. At the same time, the ability of LAs to purchase new technologies was severely curtailed by drastic cuts to local government budgets under the politics of austerity following the 2008 financial crisis. According to the Association of Directors of Adult Social Services (ADASS), LAs in England cut funding for ASC by GBP 7.7 billion in real terms between 2010 and 2019 [13]. Many LAs are now in an even more precarious financial position as a result of the novel coronavirus (COVID-19) pandemic. The large number of deaths in care homes, combined with the decisions of many families to delay moving older relatives into residential care, has further impacted the already shaky system through which private care home residents cross-subsidise state-funded residents [14].

With little guidance on increasingly specialised technologies, and no strategic vision for the future of ASC forthcoming from central government, LA technological implementations beyond traditional telecare have remained largely piecemeal [11]. LA employees working in ASC often lack expertise in complex technologies, and among those I interviewed, approaches to emerging digital care technologies largely depended on the technical capabilities and knowledge of the LA team or even individuals, as well as a greater or lesser reliance on care technology or telecare companies for information and advice. This has led to the sporadic small-scale piloting of new technologies, usually without large scale adoption due to the lack of funding available to do so, as well as the lack of a joined-up evidence base for their presumed benefits. As one LA interviewee admitted, “in all honesty I feel, it feels a bit overwhelming, the whole digital landscape”.

The challenges presented by the digital switchover, due to take place by 2025, when analogue telephone services across the U.K. will be switched off and replaced by digital internet protocol (IP) networks, help to illustrate these points. The broad national wave of telecare that the Preventative Technology Grant firmly established was analogue; these devices will become inoperable after 2025 unless they are retrofitted to work digitally. There is no national strategy or fund like the Preventative Technology Grant to help plan and pay for this work, or for the replacement of traditional telecare devices with a more innovative alternative. As Hamblin notes [10], the cost of this work is estimated at between GBP 150–300 million. As a result, while some LAs are fully prepared for the switchover, others at time of writing have no plan for how to deal with this fast-approaching deadline, or are struggling with the substantial expense involved in upgrading the equipment.

A specific set of circumstances conducive to the use of smart speakers has emerged from the parlous state of LA finances, particularly in respect of ASC, combined with an aporetic approach to care technology often dependent on individual expertise or contacts within and between LAs, a longstanding absence of national political leadership on ASC in general and care technologies in particular, and an increasingly complex care technology landscape. LAs are compelled to consider new technologies as one of the few possible solutions to further reducing the cost of care, as well as to deal with growing workforce shortages likely to be exacerbated by Brexit. There is a widespread belief among LAs that telecare and digital technologies will enable them to cut the cost of ASC: the ADASS Budget Survey 2019 noted that 96% of directors of adult social care saw the use of assistive and communications technology as quite or very important for making financial savings [13].

While there appeared to be considerable and widespread confusion about the range, and (cost) effectiveness of emerging digital care technologies among the people at LA governments involved in ASC whom I interviewed, a striking finding was their near-total convergence around the trialling and use of smart speakers and tablets with voice controlled virtual assistants. The first trial of such devices appears to have taken place in Hampshire County Council. In 2018, following an award of GBP 50,000 of funding by the Local Government Association and NHS Digital through the Care and Health Improvement Programme’s (CHIP’s) Local Investment Programme—one of the few central government funds available for the development and trialling of technology at the local level—Hampshire piloted Amazon Echo devices with customised Alexa Skills connected to various household networked devices, aiming to support independent living and provide more personalised care for 50 clients [15,16]. Since then, similar trials have rapidly emerged across England. Although Vogl et al. note that virtual assistants cannot replace the need for hands-on human care for many tasks [17], their use is usually framed around the need to save money and combat current and future care staff shortages, implying some level of substitution for human care workers—and particularly for the growing number of home care visits [16]; Hampshire County Council claimed savings of at least GBP 66,300 in its trial for 50 users [15]. The need to reduce human–human contact through the use of technologies during the ongoing COVID-19 pandemic seems to have provided a further strong catalyst for the use of devices such as smart speakers and tablets; in September 2020, the Secretary of State for Health and Social Care, Matt Hancock, announced 11,000 iPads to enable care home residents to communicate with family members while avoiding “unnecessary” in-person visits [18].

## 2. Materials and Methods

Between March and May 2020, I interviewed representatives from eight LAs in England involved in commissioning, trialling, and working with digital technologies used in the provision of ASC, as part of a wider project examining the potential of technology for sustainable care, based at the University of Sheffield’s Centre for International Research on Care, Labour and Equalities (CIRCLE). The eight interviews were semi-structured, and covered a range of topics relating to the use of digital technologies in ASC, including technologies used in the planning and delivery of care, digital infrastructure, and issues related to the use of data and analytics. Those interviewed ranged from a Director of Adult Social Services to commissioning managers; all had a significant level of decision-making authority in their LA with regard to testing, trialling, or commissioning care technology equipment. The interviews were conducted remotely via video calls, involving both one-on-one and group sessions that lasted between 30 and 60 min; group interviews were conducted in cases where the initial interviewee recommended that colleagues involved in the use of technology in ASC should also participate in order to provide further information. In all, six men and five women participated. All interviewees were asked whether they were trialling or using smart speakers or voice controlled virtual assistants in ASC, with follow-up questions about why this was or was not the case. The interviews were transcribed and then manually coded and analysed.

All subjects gave their informed consent for inclusion before they participated in the study. The study was conducted in accordance with the Declaration of Helsinki, and was approved by the University of Sheffield Research Ethics Committee (Reference Number 026350). Personal identifying data, including exact job titles, have been anonymized.

## 3. Results and Discussion

In this section, I will present and briefly discuss results from the interviews across three different areas: how Alexa has been adopted and used by LAs; ambivalent responses to Alexa from some LAs, reflecting the complexity of the care technology market; and challenges of data, information governance, and privacy presented by the use of Alexa and other voice controlled virtual assistants.

### 3.1. Adoption and Usage of Alexa

Participants in seven of the eight interviews stated that they were actively making use of Alexa-enabled devices, either in ongoing trials or, in one case, as a component of their standard telecare offering. In some cases, the LA was directly involved in developing Alexa “Skills” (a customised verbal interaction with Alexa, similar to an app on a smartphone; capitalised throughout this paper for the avoidance of confusion). These included: a Skill that prompted users to take their medicine; a Skill that helped to record and manage care tasks; a Skill to facilitate communication with caregivers by recording messages; and a Skill to connect users to a trusted LA directory of services. Other LAs made use of existing Alexa Skills not specifically related to care, such as the ability to control other smart devices and environmental controls in the home, for example lights and thermostats, in order to try to reduce the number of in-person domiciliary care visits which users required. These applications, of course, require additional hardware in the form of networked devices such as “smart” lightbulbs and thermostats, as well as an Amazon account. As one interviewee said, “I have come across examples where people have got Alexas and Echos and they set it up in the middle of the room and then gone, ‘Alexa, turn the lights on’, and then wondered why the lights haven’t gone on! They haven’t realised that… if you don’t have an account, it’s not going to be doing that.”

The Head of Technology and Digitally Enabled Care at one LA explained that Amazon’s Alexa-enabled devices were now part of its “mainstream offer” for ASC, and described the utility of Alexa, particularly for people who had little movement or those with neurological conditions, as well as how it could help a family carer leave the house while staying connected with their relative at home:

“It enables those people to live independently and it can deliver some very simple things that can make a big difference, so you know, its capability of managing the radio, helping people turn their TV on and off and all the rest of it, or change channels, communicate with loved ones. It just means that actually somebody who can’t get up and do things may be able to sort of turn their lights on and off, open their curtain, see who’s at the door, add to a shopping list even while someone’s out shopping, all those sorts of things… What we found is for those people who use it, [it’s] enough to be the tipping point between people making a choice about whether they feel they can live independently now or not.”

A Commissioning and Contract Manager at a different LA described how they were “very interested in Alexa” and were working on a new Skill in consultation with Amazon Web Services:

“We’ve got to come up with a script [i.e., for the conversation between the user and Alexa], but basically what I want them to do is, if you fall over and you can shout and you’ve got an Alexa in your house, we will come up with a script and it will then call our call centre. So, the problem is though, I don’t want them to fall down and shout ‘Help!’ and then [Alexa] say, ‘Help! The Beatles number’ [laughs]. So we’ve got to come up with a script… So we’ve got quite a few bits to overcome, but as a council we’ve worked very closely with them—Amazon and Alexa. So we’ve already put into all of the local Alexas in [city name], so if you say ‘Hey Alexa, what day does my bin get emptied?’, they’ll tell you. And so the top ten questions that we get asked as a council, we put into Alexa the answers. In the current COVID crisis, that’s been very useful.”

A third interviewee explained how their use of Alexa had gone a step further than this sharing of information about council services. They described how the local services themselves had been integrated with Alexa at a “mini pilot village of the future”, where the LA was trialling various different technologies, enabling older people to access internet-based services by voice, without having to use a computer:

“They worked with the local community and they then used Alexa as the kind of linking infrastructure. So, they worked for example with the local pub, local fish and chip shop, local shop and then our guys internally built a couple of Alexa apps where you could, the businesses could put all their menus on, build all their billing in the backend and then somebody living in [town name] could have the app, could have it on their Alexa, and say you know, ‘Alexa I want to order my lunch’, the menu would pop up, they could order their lunch and then that got delivered and all the billing just happened in the background. You know, really simple—but it allowed people who were potentially socially isolated to access a warm meal and not worry about, you know, where it was coming from or who was coming to the door, all that sort of stuff. So, we’ve done that and then we’re looking to roll that out into, into [town name] and other places at scale. It’s kind of, again it’s just facilitating what already exists but just making it that bit, that bit easier…we seem to be having fairly circular conversations with [the LA’s telecare provider] about, you know, how we embed things like Alexa as an alternative to the traditional telecare hub and what it allows people to do. I’m not sure, we’re not there yet but it’s certainly part of the discussion.”

As can be seen from these excerpts, devices such as Amazon’s Echo smart speaker have the advantages of being sophisticated and powerful, relatively cheap, already widely used and familiar, designed with a degree of accessibility and intuitive use in mind, and a growing level of interoperability with other networked digital devices aided by an open development framework. They are dynamic in the sense that additional apps or Skills are being continuously developed and made available to users, and as popular mainstream devices, they avoid the stigma of beige telecare devices specially designed for “the elderly” that inscribe negative stereotypes [19]. Voice controlled virtual assistants seem to embody a more consumer-oriented, “smarter” provision of technology to deliver on the promise of independent living, with Alexa becoming a kind of substitute caregiver or companion that can empower users who require only moderate levels of everyday support, for example controlling their phone or lights by voice, or providing a medicine reminder. At a time when ASC funding in England is in crisis, and the care technology market is more fragmented and complex than ever before, the familiarity, convenience, and low cost of smart speakers made them seem, to several interviewees, a quick and easy win to reduce council spending, particularly on home care visits.

### 3.2. Complexity and Fragmentation of the Care Technology Market

It is important to note that the 152 LAs responsible for the delivery of social care services in England are heterogeneous, with different population sizes and demographic situations, different ASC budgets, different internal organisational structures, and different levels and types of technological expertise. The three interviewees quoted above were employed by LAs that had specialised roles specifically devoted to exploring the application of technology to ASC. Others had no dedicated resource and relied on external contractors or strategic corporate partners to advise on care technology, or were following suit in a rather vaguer fashion with no clear strategy for training or implementation, or for how these devices would fit within the existing framework of ASC services. As the ASC Assistive Technology Lead at a different LA explained:

“We just, we’ve got some voice activated assistants and Alexas and Googles coming to try and experiment with and to, I guess, allow staff to have a go with as well as to show to people, this is what can be achieved with them. I have, I have concerns about data so I’m very, very wary about prescribing them to people, but I think allowing people to see what they look like and see what they can do and try for themselves and then if they decide to go and buy them, then I think that’s my stance at the moment, but I’m kind of working through that.”

An ASC Commissioning Manager at one of the largest LAs in England stated that they were not using voice-controlled assistants, although the rationale for this decision seemed somewhat unclear:

“We haven’t used Alexa, there has been a bit of, not resistance, but a bit of hesitation really, because, there are good opportunities there, but I can remember reading an article, well I read two articles in the same week, one sort of saying, ‘Oh, you know, this is the way to go’, and then I’ve read another one saying, ‘Actually there’s some people with dementia where this is totally inappropriate, where it’s frightened them to death because this machine’s talking to them.’”

The relative lack of detailed technical knowledge of these two interviewees is indicative of the rapidly changing marketplace in care technologies in the U.K., the increasingly complex and fragmented set of products and services now available, the lack of evidence base for their effectiveness, outcomes, or value for money, and the absence of any centralised independent authority that can provide specialist advice on care technology to LAs [11]. The closest thing to such a centralised advisory body is the Technology Enabled Care Services Association (TSA), the main industry body for telecare and telehealth in the U.K., which lobbies government on issues of technology in care, operates a quality standards framework certification scheme, and maintains a close relationship with the Association of Directors of Adult Social Services (ADASS). However, as an industry body, its interests lie with its members, which are the companies selling equipment and services to LAs, and therefore it cannot be viewed as a disinterested party. As a result, LAs are left to navigate the emerging digital tech landscape as best they can [11]. Many interviewees mentioned that their sources of information on new care technologies came from a mixture of social media, contact with other LAs and companies, direct email advertising, and exhibitions such as that held during the ITEC conference mentioned above. One interviewee, a deputy director of commissioning, introduced himself to me at the 2019 ITEC conference as his LA’s “Chief Officer for weird shit”—it was meant as a joke, but is also illustrative of the still-exotic image of digital technology for many working in ASC in local government. Many contacts I made at LAs, including the deputy director, repeatedly described themselves to me as “amateurs” with an “interest” in technology, rather than specialists, even when they were the main advocate for or expert in technology for ASC at their council. In this situation, it may seem natural to turn to the most familiar and convenient everyday consumer technologies, including voice assistants.

### 3.3. Data, Information Governance, and Privacy

The absence of authoritative independent advice on care technologies available to LAs, coupled with the broken funding system for ASC exacerbated by the COVID pandemic, had scuppered the possibility of using the 2025 digital switchover as an opportunity to substantively improve existing telecare infrastructure. As the Chief Executive of a national care innovation centre I interviewed put it:

“The sad fact is that across significant waves of the U.K., people are talking about replacing like for like, in other words, just simply replacing an analogue service with a digital version of the same, and I think personally that is very wasteful—it’s wasteful of the opportunity, but it’s wasteful in terms of money as well. The beauty of moving to digital is that currently most home and mobile health digital services sit on digital platforms. So you can see you’ve now got the opportunity to actually integrate health and social care responses into single packages, if you then move to this coordinated next generation digital service model.”

However, many of my LA interviewees argued that although the digital switchover was likely to be a missed opportunity, new versions of telecare would emerge in the near future. As one interviewee, who worked as a self-described “tech champion” across her LA ASC team and the local NHS Clinical Commissioning Group (CCG), explained:

“I think we’ll start to see that over the next five years, particularly as we shift from analogue onto digital, that the telecare services of the past will have to morph into what the future looks like, using much more modern technologies and not being too restrictive. We’ve got to have an open source platform I think to be able to do some of that, because otherwise we’re never going to really deliver personalised care, it’s going to be the same old pull cord [i.e., traditional telecare system], a falls belt around your waist, and a falls mat, and actually there’s so much more out there.”

Several interviewees told me that they were looking at voice controlled virtual assistants as a potential replacement for traditional telecare systems, or as a new interface to established telecare call centres. The deputy director of commissioning mentioned above suggested that if Amazon put a SIM card into their Echo devices, they would effectively destroy the traditional telecare industry: “If I was one of those big traditional telecare companies like Tunstall, I’d be slightly nervous. Why would you need a specialist bit of kit when you can say, ‘Alexa, I’ve fallen over’, ‘Alexa, when do I need to take my medicine?’” Another interviewee said, “ultimately one of the things we’d like to do is: can we, through our telecare, link into things like Alexa and Echo, rather than build separate systems? That’s a long way down the road but that would be our mission”.

One obstacle to this vision was that Alexa devices were usually not being offered directly by LAs to ASC service users. This was partly due to the cost of doing this at a large scale. As one interviewee explained, “the potential of WhatsApp is huge and, similarly I think Alexa and so on. But the big question for us, to be harsh about it, is: who pays for it?” LAs tended to encourage ASC users or their families to purchase an Alexa device in order to take part in local trials, and in some cases, they found that a number of local care recipients already had such devices. This arrangement also had the important benefit of enabling LAs to sidestep highly complex data governance issues, because the user agreement for the device was between Amazon and the customer—the LA itself was not directly involved. As another interviewee, who was a former LA Data Protection Officer, put it, when I asked about data governance in relation to Alexa, “You know, global organisations like Amazon… that is a bit of a minefield.” But in the same (group) interview, the Director of Adult Social Services at the same LA quickly added:

“To some degree, people are out there sharing their data with these four large companies, aren’t they? Google, Amazon, Microsoft and Apple. So, to some degree, we’re just relying on their consumer relationship, we’re not getting in the way of it, for me it’s part of the answer—it’s utilising ordinary universal things that connect families with vulnerable people together, so they can check on one another and care for one another. Without us coming into the equation necessarily. It is about some of these, some of the Skills, some of the other things that other people want to develop really. We don’t have to always do that ourselves I think.”

Nevertheless, there were some data governance issues associated with LAs developing their own Alexa Skills or using Alexa devices for ASC delivery. Again, there seemed to have been little or no guidance in these areas from central government for LA ASC departments. The Head of Technology and Digitally Enabled Care at a LA described by other interviewees as a national leader in care technologies said:

“I was really keen that we tried to develop a, a, Skill that was literally about, you know, could we link into the diaries of care agencies to say, you know, if I said, ‘Where’s my carer?’ it’d interrogate the rota system and say, ‘Oh, so and so is coming, they have been delayed’ or whatever. But… we couldn’t do that because of all sorts of information governance, we were stripped of our capabilities of doing that. And I think there are some things that we as a society need to be having a conversation about around data governance, about what is and isn’t useful, or what we should and shouldn’t be thinking about doing going forward… there is a discussion to be had nationally about information governance and stuff like that.”

They added, “It’s one of those areas where you know we’re still learning these sorts of things but it’s this issue of us [i.e., LAs] becoming the curators of knowledge as opposed to deliverers of services.”

Issues of information governance extended to the question of where the data collected by these devices would end up. An ASC Commissioning and Contract Manager said, “I think [data] is going to become more and more [valuable]… In a world where the councils have got no assets but they have got access to data, I think it will become more important.” But as the Chief Executive of a national care innovation centre put it, “Oh god… don’t get me wrong, [Alexa] does some very interesting things. The issue is that as soon as you use Alexa, Amazon has your data. Amazon stores that data in the United States, and Amazon monetises that data for its personal use. That is unacceptable to me and I think it would be unacceptable to the vast majority of people if they were actually aware that that is what is happening.”

While literature on the datafication of the welfare state in the U.K. and beyond has, to date, focused on concerns such as transparency, bias and lack of redress in algorithmic decision making, and the danger of digitally entrenching inequality [4,5,6,7], the case of the Alexafication of ASC highlights overlapping but slightly differing issues. These include data governance and privacy, the digital divide, and inequalities of access, as well as questions about adherence to technical quality and reliability standards such as those required by law for telecare equipment—concerns that have also been discussed by Hamblin in the broader context of the possibilities and challenges presented by a new generation of digital technology-enabled care services against the backdrop of the upcoming digital switchover [10]. Based on the results presented above, the main contribution made by this paper to these discussions, as will be explored in the next section, is highlighting the risk of increasing public sector reliance on precarious corporate infrastructures for the wellbeing of older adults under the care of LAs, and the misalignment between local government ASC and global technology corporations, as well as suggesting the way in which such corporate technological entanglements with adult social care delivery may transform the role of LAs themselves.

## 4. Conclusions

In the prolonged absence of national political leadership in the U.K. in proposing solutions to the myriad challenges of adult social care, relatively cheap, ubiquitous, and approachable consumer technologies such as Alexa seem, based on the interview data presented in this paper, to be fast becoming one of the most important elements of a de facto strategy among LAs for a technologized future of care less centred on in-person human interaction. However, as new, more complex care assemblages come to include a fragmented patchwork of generic consumer electronic devices, yoking together a fast-moving and global consumer technologies marketplace characterised by the epithet “move fast and break things”, with the slow commissioning practices and long telecare contracts of local government, they introduce both new opportunities and new areas of risk, uncertainty, and fragility that could redefine the future role of LAs.

As more ASC services move online, new challenges are emerging [10]. The increasing digitisation of care services in broad terms is likely to exacerbate an already significant digital divide, jeopardising equality of access. According to a 2019 study by the Oxford Internet Institute, half of adults over the age of 65 in the U.K. do not use the internet, and there are substantial rural–urban differences in the coverage and quality of broadband, 4G, and now 5G supply [20]. While “traditional” analogue telecare requires only a fixed phone line, and can still be used during power cuts, smart speakers, which are not currently subject to the same safety standards as telecare devices, are vulnerable to power cuts, internet outages, network slowdowns, and internet or Wi-Fi equipment failure, not to mention hacking and cyber-attacks. If the future use of smart speakers in place of traditional telecare services entails the replacement of often locally located call centres with natural language processing speech recognition, the availability of services may also increasingly depend on citizens with regional accents or speech difficulties making themself intelligible to the device, configuring themselves to the smart speakers. There are also questions about both the ethics and the long-term financial sustainability of paying tax-avoiding international technology companies such as Amazon to deliver public services. Amazon Echos are cheap and ubiquitous in part because, according to lawmakers in the U.S. House Judiciary subcommittee in October 2020, Amazon has been selling them below cost price [21]. It was able to do so in turn because it has long avoided paying a significant amount of corporation tax—for example, the company paid only GBP 14 million in U.K. corporation tax on GBP 2.3 billion of U.K. sales revenue in 2018 [22].

Moreover, global technology companies selling generic consumer products and services may not be responsive to the specific needs of LAs or ASC. The Deputy Director of Commissioning at one LA talked about a major project he conducted with Samsung, which involved providing 300 older people in the area with smart watches to monitor various types of data, including the number of steps walked, heart rate, sleeping patterns, and usage of household appliances. He described the results of the project themselves as “a bit meh… It’s not massively worked”, but tellingly also pointed out difficulties in technical support: “I’ll be honest, we learnt a lot more from how it didn’t work than how it did… Samsung upgraded [i.e., updated the software in] all their phones worldwide, you wouldn’t have even noticed, it was a little glitch—you know, ‘Click here’ type thing—but it didn’t speak to this system and when we pointed out [that the smart watches] had all stopped working, Samsung’s answer was, ‘Oh if you just go round and visit people you can talk them through how to change it.’ But we can’t go visit 300 older people and take them through a script”. The same interviewee also described a meeting with Amazon representatives that likewise suggested a mismatch between the context and needs of English ASC and the approaches of technology companies: “Amazon…proposed to me that they could do little very tiny recording devices that we could attach to social workers so all their conversations were recorded and stayed in the cloud, and then they could use that both for putting down [i.e., transcribing] onto files but [also] develop a sound cloud for me about what the key issues were that were coming up… I just quite like the idea of me going back to my head of social work and going, ‘I’ve just done this agreement with Amazon that we’re going to record all social care workers and oh, by the way, they’re going to own it!’ [laughs]”.

Similarly, the use of ready-made consumer technologies seems to run counter to the growing awareness of the importance of practices of co-production, co-design, user-centred design, or participatory design, in the development of care technologies [23]. Despite the ubiquitous rhetoric of co-production in ASC discourse among LAs in England, it is unclear whether an approach that uses Alexa involves substantive participatory co-production on the part of older end users or other stakeholders, because they are dealing with a finished commercialised product, and the co-production of new Skills, (if it happens at all, given the complexity and data governance issues involved), can only take place within the development environment provided by Amazon.

In introducing Amazon’s products and services into what one interviewee quoted above described as “the linking infrastructure” of local services for older people, and in weaving Alexa’s technological ecosystem into the delivery of aspects of ASC, there is also a significant risk that LAs will move towards a position of dependence on the technological infrastructures of global corporations. The slide towards the Alexafication of ASC seems an approach driven more by crisis decision-making by LAs in a situation of precarity and desperation than by any empirical evidence that it provides the best outcomes or even long-term value for money for citizens using council services, or addresses the deeper problems facing the ASC sector. Turning to convenient (rather than co-designed) consumer devices may seem the easiest option for LAs, even if it involves effectively privatising public services by the back door. It may appear to be the only way to resolve the fundamental mismatch between LA responsibilities for delivering ASC in discrete local silos, the growing imperative to plan and implement interoperable, highly complex connected digital systems, and inadequate funding—by becoming local mediators between tech companies and consumers, or as an interviewee quoted above put it, “curators of knowledge not deliverers of services”. This is particularly the case as the market for digital care technologies becomes increasingly fragmented, and issues of data governance that arise from such products become ever-more complex, while LAs often lack the technical knowledge and expertise to navigate them.

It is worth recalling that Alexa has only existed for six years, and there is no guarantee either that it will still be around for another six, or that other smart speaker hardware or software will continue to be supported into the future, or that user data will not be sold to other companies or used for new purposes. ASC tends to require stability over many years in order to provide a predictable service to a planned budget; as demonstrated by the digital switchover in 2025, even changes known about for years can still prove highly disruptive. Indeed, the digital switchover appears almost as a symbolic turning point, where connections to a more stable analogue world are literally being cut in favour of a faster-paced, more precarious, and riskier digital world.

In piloting new consumer technologies such as smart speakers, some LAs seem to be piloting new versions of what a LA can and should be in the future, in relation to its citizens. The use of generic consumer technologies seems to be a key part of a marshalling of disparate affordable resources to reshape a fundamentally smaller, seemingly more agile, LA, and in fact several have started to adjust their internal structures, creating “innovation hubs”, and negotiating new kinds of relationships and strategic partnerships with technology companies. In this future vision of care, LAs may find their role becoming closer to that of an app developer and data broker than a commissioner of social care services, to some extent mediating and facilitating the monetisation of a consumer relationship between powerful technology companies and citizens for the purposes of care. In this scenario, public services such as ASC would likely be increasingly incorporated into a supranational commercial app ecosystem controlled and owned by monopolistic global technology companies—the reverse of the previous system where the government created the national commercial ecosystem for private companies to provide services—with a concomitant loss of power and control, including over what is emerging as one of local government’s most valuable remaining assets: citizen data.

These findings suggest an urgent need for a national social care technology strategy for England, including sufficient funding for LAs and an independent national advisory service that can provide expertise and guidance on the effectiveness of care technologies based on the available evidence, as well as on the long-term implications of their adoption. They also suggest the need for further national public debate about the consequences of engaging with global technology companies for the provision of essential public services, about the types of ASC that citizens want, and how this system will be paid for and managed.

It is important to highlight the limitations of this study. The sample of interviewees represents only around 5% of English LAs (eight out of 152), although the fact that the overwhelming majority were using Alexa is strongly suggestive of a broader trend, and this paper is in any case not intended to survey the use of Alexa across the whole of England. Important future lines of research in this area will include: understanding how representative this study is of the wider situation of virtual assistant use in ASC in England, the U.K., and beyond; gaining a better understanding of how and on what basis LA commissioners and other institutional intermediaries and gatekeepers between taxpayers, corporations, and care recipients make decisions about the purchasing or trialling of care technologies, and how they work with care providers and care recipients to implement them; and how the specific risks associated with Alexa and other voice controlled virtual assistants used in ASC that have been identified in this paper unfold over the coming years.

## References

[1] UK Department of Health and Social Care NHS Health Information Available through Amazon’s Alexa. www.gov.uk/government/news/nhs-health-information-available-through-amazon-s-alexa.

[2] Walker A. (2019). NHS Gives Amazon Free Use of Health Data under Alexa Advice Deal. The Guardian.

[3] Brown S. (2019). Partnerships between health authorities and Amazon Alexa raise many possibilities–and just as many questions. CMAJ.

[4] Rudschies C., Rieder G. Notes on the political economy of welfare AI. Proceedings of the EASST/4S Conference.

[5] Dencik L., Kaun A. (2020). Datafication and the welfare state. Glob. Perspect..

[6] Yeung K. Algorithmic government: Towards a new public analytics?. Proceedings of the ThinkBig Workshop.

[7] Alston P. (2019). Report of the Special Rapporteur on Extreme Poverty and Human Rights.

[8] Madjaroff G. (2018). Supporting caregivers and care recipients after the onset of cognitive impairment with home based technology. Innov. Aging.

[9] Sriram V., Jenkinson C., Peters M. (2020). Carers’ experience of using assistive technology for dementia care at home: A qualitative study. BMJ Open.

[10] Hamblin K. (2020). Technology and social care in a digital world: Challenges and opportunities in the UK. J. Enabling Technol..

[11] Wright J. (2020). Technology in Social Care: A Review of the UK Policy Landscape.

[12] European Commission (2010). ICT and Ageing: European Study on Users, Markets and Technologies Report. https://ec.europa.eu/digital-single-market/en/news/ict-and-ageing-european-study-users-markets-and-technologies.

[13] Association of Directors of Adult Social Services (2019). ADASS Budget Survey 2019.

[14] Jarrett T. (2018). Social Care: Care Home Market–Structure, Issues, and Cross-Subsidisation (England).

[15] Local Government Association (2018). Local Investment Programme: Hampshire County Council–Using Voice-Activated Home Audio Speaker to Promote Independence and Wellbeing. https://www.local.gov.uk/sites/default/files/documents/Hampshire%20County%20Council%20LIP%20Case%20Study.pdf.

[16] Taylor S. (2018). BBC Daily Politics Show. www.youtube.com/watch?v=YL-nQGPxc68&feature=youtu.be.

[17] Vogl T.M., Seidelin C., Ganesh B., Bright J. (2020). Smart technology and the emergence of algorithmic bureaucracy: Artificial intelligence in UK local authorities. Public Admin. Rev..

[18] Stubley P. (2020). Coronavirus: Government to Give 11,000 iPads to Care Homes So Residents Can Speak to Families Tablets Will ‘Stop Spread of Virus by Reducing Unnecessary Visits’, Say Officials. The Independent.

[19] Neven L., Peine A. (2017). From triple win to triple sin: How a problematic future discourse is shaping the way people age with technology. Societies.

[20] Blank G., Dutton W. (2019). Perceived threats to privacy online: The internet in Britain. Oxford Internet Survey 2019. SSRN Electron. J..

[21] Palmer A., Novet J. (2020). Amazon Bullies Partners and Vendors, Says Antitrust Subcommittee. www.cnbc.com/2020/10/06/amazon-bullies-partners-and-vendors-says-antitrust-subcommittee.html.

[22] Butler S. (2020). Amazon Accused of Handing over ‘Diddly-Squat’ in Corporation Tax. The Guardian.

[23] Fischer B., Peine A., Östlund B. (2020). The importance of user involvement: A systematic review of involving older users in technology design. Gerontologist.

